# Concentrated Ionic Fluids: Is There a Difference Between Chloride‐Based Brines and Deep Eutectic Solvents?

**DOI:** 10.1002/anie.202311140

**Published:** 2023-10-11

**Authors:** Guillaume Zante, Christopher E. Elgar, Katherine George, Andrew P. Abbott, Jennifer M. Hartley

**Affiliations:** ^1^ University of Leicester College of Science and Engineering Leicester LE1 7RH UK

**Keywords:** Brines, Deep Eutectic Solvents, Electrochemistry, Ionic Liquids, Oxidation

## Abstract

Deep Eutectic Solvents (DESs) have been lauded as novel solvents, but is there really a difference between them and concentrated aqueous brines? They provide a method of adjusting the activity of water and chloride ions which can affect mass transport, speciation and reactivity. This study proposes a continuum of properties across concentrated ionic fluids and uses metal processing as an example. Charge transport is shown to be governed by fluidity and there is no discontinuity between molar conductivity and fluidity irrespective of cation, charge density or ionic radius. Diffusion coefficients of iron(III) and copper(II) chloride in numerous concentrated ionic fluids show the same linear correlation between diffusion coefficient and fluidity. These oxidising agents were used to etch copper, silver and nickel and while the etching rate increased with fluidity for copper, etching of silver and nickel only occurred at high chloride and low water activity as passivation occurred when water activity increased. Overall, brines provide a high chloride content at a lower viscosity than DESs, but unlike DESs, brines are unable to prevent passivation due to their high water content. The results show how selective etching of mixed metal waste streams can be achieved by tuning chloride and water activity.

## Graphical abstract

Conductivity, viscosity, water, and chloride content were measured for brines and aqueous DESs. Low viscosity brines favour copper etching due to higher mass transport. Low water content DESs promote etching for silver and nickel.

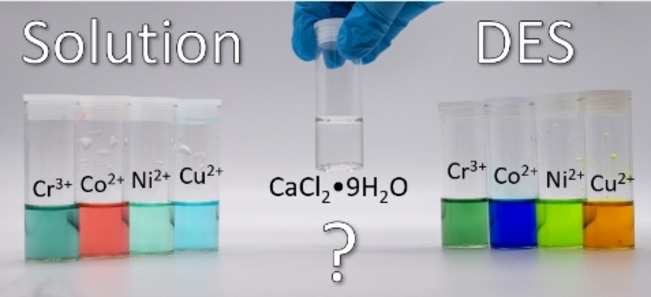

## Introduction

Ionic liquids (ILs) and deep eutectic solvents (DESs) are lauded as novel solvents for metal processing due to their low water content and the ability to control speciation.^[1]^ Recent work has confirmed that metal speciation and electrochemistry is controlled by the solvent anion,^[2]^ and that the presence of small amounts of water (<10 wt %) does not affect the species present.[^3^, ^4^, ^5^] The original definitions of ionic liquids and DESs[^6^, ^7^, ^8^, ^9^] do not appear to be helpful and a recent overview suggested that there is a continuum spanning ionic liquids, protonic ionic liquids, DESs, salt hydrates and brines.^[10]^ The continuum exists due to the equilibria between the metal cation and ligands in the liquids which varies depending on the Lewis acidity of the metal and the Lewis basicity of the neutral (L) and anionic (An^−^) ligands.

(1) $${\left[ML_{x}\right]}^{a+}\vec{\leftarrow }[M{An}_{y}L_{x}{]}^{a+/-}\vec{\leftarrow }{\left[M{An}_{y}\right]}^{a-}\;$$

This equilibrium moves to the right for ionic liquids and to the left for brines and is in the middle for salt hydrates and DESs. The exact position depends on the Lewis acidity of the metal and the concentration of the species present. Thus, this whole area is best described as a concentrated ionic fluid rather than the individual historic labels which have previously been used to describe specific liquid compositions. This equilibrium can be used beneficially for selective metal processing. For example, when using iodine to oxidise copper, nickel, and gold, in a DES comprised of ethylene glycol (EG) and choline chloride (ChCl), the presence of water increases the oxidation rates of copper and gold due to the decreased viscosity, but decreases the dissolution of nickel due to passivation.^[11]^ Neutron scattering studies showed that the DES nanostructure is conserved until a relatively high content of water (42 wt.%) is added to the DES, with water forming nanostructured domains within the solvent.^[12]^ Beyond this point, the DES turns into an aqueous solution of hydrogen bond donors and hydrogen bond acceptors. This switch between DES/IL with water dissolved in it and an aqueous solution of DES/IL components has also been observed for other types of DES and IL mixtures with water, via spectroscopic methods. The critical water content to cause changes in metal ion[^3^, ^4^, ^6^, ^13^] or halide^[11]^ speciation was 30–45 wt % in all cases described so far.

Chloride brines have been used in a range of different applications, such as chemical biology in buffered saline solutions,[^14^, ^15^] mineral processing,[^16^, ^17^, ^18^] and E‐waste recycling.[^19^, ^20^] Chloride salts were selected for this investigation because chloride is common in both natural brine sources[^21^, ^22^] and the concentrated brines produced after desalination of seawater,[^23^, ^24^, ^25^] which can contain over 60 g/L of sodium chloride.^[26]^ While brines formed using other types of cation‐anion combinations are available, from a practical point of view, chloride brines are more likely to be used in metal processing due to their large‐scale availability and a better understanding of metal behaviour in chloride media. The physical properties of these chloride brines have been widely studied, and they generally have lower viscosity and higher conductivity values compared to DESs.^[27]^

The current study aims to investigate the similarities between chloride‐based DESs and the corresponding aqueous brines using the same salts. The two types of brine investigated; choline chloride and calcium chloride hexahydrate. Both have been used in DES formulations but they have different sizes and charge densities which should affect both the anion and water activities. Calcium chloride hexahydrate is a particularly interesting source of chloride as it is available in large amounts, as it is a waste product from the Solvay process^[28]^ and, contrarily to lithium or magnesium, is not a critical raw material.

The physical properties of these brines will be characterised and compared to a DES formed from choline chloride and ethylene glycol with different amounts of additional water. The diffusion coefficients of the Cu^I^, Cu^II^, Fe^II^ and Fe^III^ species present will be investigated and then applied to the etching of copper, silver, and nickel to identify whether etching rates are controlled by mass transport of the oxidising agent or chloride concentration of the solvent.

## Results and Discussion

### Characterisation of the physicochemical properties of the brines

**Figure 1:**
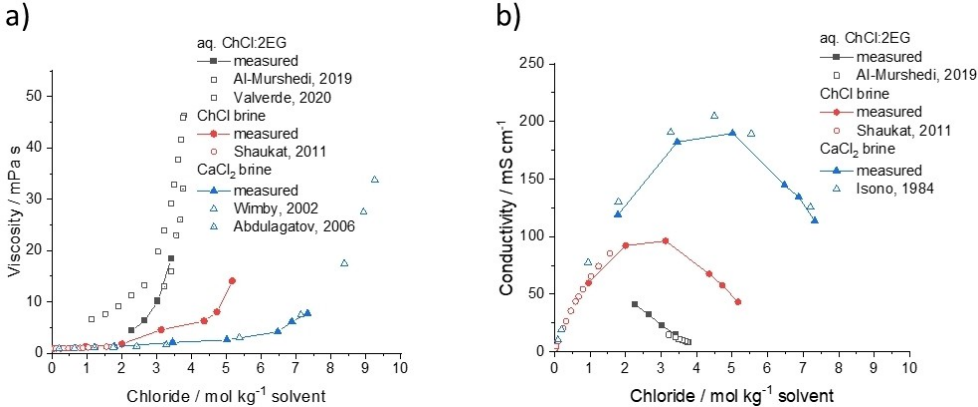
a) Viscosity, and b) conductivity as a function of chloride content in moles chloride per kg of solvent. Temperature is 25 °C. Literature values obtained from Shaukat,^[29]^ Al‐Murshedi,^[3]^ Valverde,^[13]^ Wimby,^[30]^ Abdulagatov,^[31]^ and Isono.^[35]^ Lines are used to guide the eye.

Table S1 shows the effect of salt‐to‐water ratio on the viscosity and conductivity of ChCl and calcium chloride hexahydrate (CaCl_2_ ⋅ 6H_2_O) brines, in comparison to aqueous solutions of a DESs formed from ChCl with two molar equivalents of EG. The viscosity values are presented as a function of chloride concentration (Figure 1a) and it can be seen that viscosity increases with increasing chloride content (decreasing water content), as expected. Both of the chloride brine systems correspond with literature values.[^29^, ^30^, ^31^] However, when considering the aqueous DESs, each literature source reports a range of different viscosity values.[^3^, ^13^, ^32^] This variation is likely due to a combination of different purity chemicals and different solvent preparation methods, as both ChCl and EG are hygroscopic. In general, the water content in the as‐prepared DES is in the range of 1–3 wt % water. Even so, viscosity of the DES systems remains higher than either of the brines at any given chloride concentration due to the presence of the EG and the decrease in molar free volume.[^33^, ^34^]

The effect of chloride concentration on solvent conductivity is presented in Figure 1b. In all three systems investigated, conductivity increases with increasing water content due to the corresponding decrease in solvent viscosity and increase in ionic mobility, up to a maximum value between the salt : water molar ratios of 1 : 10 and 1 : 20. This is followed by a decrease in conductivity for the 1 : 50 systems due to their lower ionic strength. The conductivity values for the CaCl_2_ ⋅ 6H_2_O brines are consistently over double the values for the ChCl brines. While the CaCl_2_ ⋅ 6H_2_O brines will have higher ionic strength than the ChCl brines, there will also be a contribution to conductivity from ion mobility. For example, the cation in the CaCl_2_ ⋅ 6H_2_O brine will be a hydrated highly symmetrical small Ca^2+^ ion, whereas in the ChCl brines, the cation is a bulky asymmetrical choline cation with poorer mobility. The aqueous DES solutions have the lowest conductivity values, likely due to the much higher solvent viscosities at any given chloride concentration.

**Figure 2:**
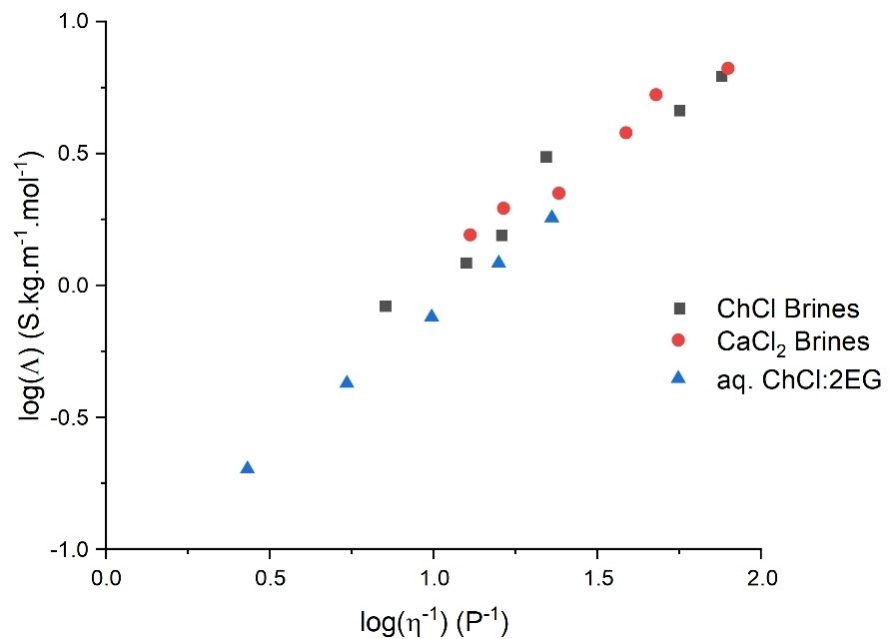
Plot of molar conductivity as a function of fluidity for the different solvents. Temperature is 25 °C.

To understand the mechanism of ion mobility it is helpful to analyse the molar conductivity of these fluids. Numerous studies have shown that there is a linear correlation between molar conductivity and fluidity of the solvent. This has mistakenly been ascribed to the so‐called Walden Rule which was originally observed for non‐aqueous solutions of electrolytes at infinite dilution.^[36]^ It has subsequently been proposed that the mechanism of charge transport is actually limited by the availability of holes which are of suitable dimension for the ions to move into.^[37]^ Figure 2 shows a plot of molar conductivity vs fluidity for all of the concentrated ionic fluids studied here. It is important to note that all liquids display the same linear correlation irrespective of the size of the cation or the size of the neutral ligand (water or EG) and even more surprisingly, the charge on the cation. An extended form of this plot is found in the Supporting Information as Figure S8, showing that this trend is also valid for other cation‐anion combinations found in the literature. Additionally, literature data for different molar ratios of ChCl and EG are included,[^33^, ^38^] and it can be seen that these systems also follow the observed trend, despite the lack of water present. It can be concluded that irrespective of the system studied the molar conductivity is limited by the fluidity of the liquid. This shows why the continuum idea of concentrated ionic fluids spans ionic liquids, DESs and brines. It should be noted that the most dilute brine used in this study is still >1 M chloride ion concentration.

### Diffusion coefficients in brines and DESs

To extend this idea of a continuum of properties across concentrated ionic fluids, the rates of chemical dissolution of metals was investigated using redox catalysts in a range of brines and DESs. The diffusion coefficients for two redox catalysts, Fe^III^ and Cu^II^, were determined using cyclic voltammetry. The diffusion coefficients for Fe^II^, Fe^III^, Cu^I^, and Cu^II^ species in the choline chloride and calcium chloride brines are presented in Table S2, along with an aqueous DES series. The cyclic voltammograms at different scan rates are presented in Figures S1–6, and the Randles–Sevcik plots are shown in Figure S7. The diffusion coefficients of all species increase with increasing water content, as would be expected from the decrease in viscosity and corresponding ease of mass transport.

In the aqueous ChCl : 2EG systems, it can be seen that up to 30 wt % water (molar ratio DES: 6.3H_2_O), the CVs for Cu^II/I^ behave reversibly, but at 40 wt % water (molar ratio DES: 9.8H_2_O) the electrochemistry changes with scan rate (Figure S1). This is likely due to the mixture approaching the limit of DES dilution,^[12]^ and the instability of Cu^I^ in aqueous systems. In the ChCl brine systems, similar quasi‐reversible behaviour is present in the most dilute 1 : 50 system, with deviations from linearity seen in the Randles–Sevcik plots at the highest scan rates, which could be due to the decreased chloride content resulting in destabilisation of the Cu^I^ species causing precipitation of CuCl at the electrode surface. This is not the case for the iron species. The redox behaviour of the Fe^III/II^ couple appears unaffected by water content (Figures S4–S6), likely due to the comparatively higher stability of both oxidised and reduced species within the timescales of these experiments.

**Figure 3:**
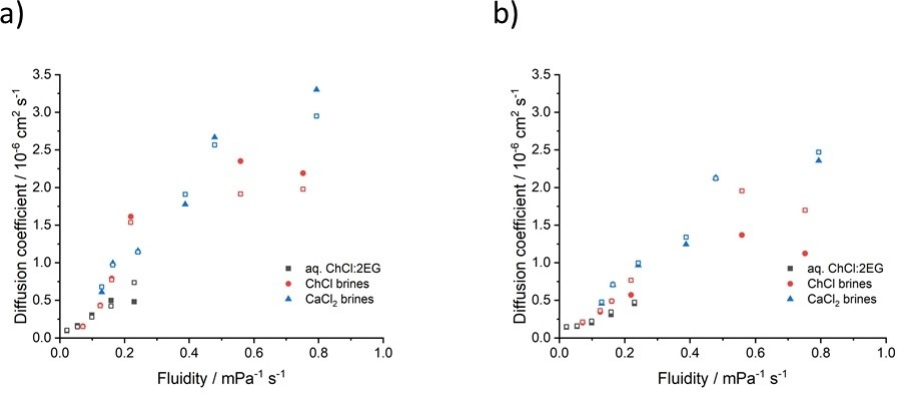
Diffusion coefficients for a) copper and b) iron ions as a function of solvent fluidity in aqueous choline chloride, calcium chloride, and ChCl : 2EG systems. Filled symbols are Cu(I), Fe(II) and hollow symbols are Cu(II), Fe(III).

Critically, plotting diffusion coefficients as a function of fluidity (Figure 3) shows that there is little difference in diffusion coefficients of the different copper or iron species between the chloride brines at the same viscosity, even though there are different charge densities on the different species, and the relative size of the ionic complexes vary slightly. In addition, the chloride concentration and ionic strength of the different solvents does not appear to have an impact, except in the most dilute systems containing choline chloride.

This means that metal etching rates when using Cu^II^ or Fe^III^ as oxidising agents in these different solvents should be directly linked to mass transport of the oxidising species (i.e. viscosity), assuming no passivation of the metal substrate or poor diffusion of the oxidised metal complexes. Hence, it would be predicted that the fastest etching should be in the second‐most dilute systems of 1 : 20 chloride salt: water and ChCl : 2EG 30 wt % water (molar ratio DES: 6.3H_2_O) when using CuCl_2_ and in the most dilute systems when using FeCl_3_ as oxidising agents.

### Metal etching in brines and DESs

The etching of copper, silver, and nickel was carried out using CuCl_2_ and FeCl_3_ as oxidising agents. These metals were selected because they should proceed spontaneously in both aqueous and DES systems from their redox potentials. Additionally, the Cu(II) and Fe(III) species present in these chloride‐based DESs and brines are already well‐characterised via spectroscopy.[^3^, ^13^, ^20^, ^39^, ^40^] In both cases, each molar equivalent of oxidising agent will remove one electron from the target metal, so for a general process, x moles of Cu^II^ or Fe^III^ will be required per oxidation state number of the oxidised form of the target metal:

(2) $$M+{xCu}^{II}\to M^{x+}+{xCu}^{I}\;$$

(3) $$M+{xFe}^{III}\to M^{x+}+{xFe}^{II}\;$$

The hypothesis was that metal etching rates should be dependent on mass transport of the oxidising agent to the metal surface, rather than the source or concentration of chloride, or the type of oxidising agent itself. If etching rate is related to mass transport alone, then the metals should have similar etching rates when the brines and DESs have similar viscosities. If the metal etching rate is related to chloride concentration, then it would be expected that etching should be fastest in the calcium chloride systems. If the etching rate follows a curved profile, then a combination of mass transport of the oxidising agent and the presence of insoluble species forming in high water environments is expected.^[40]^

**Figure 4:**
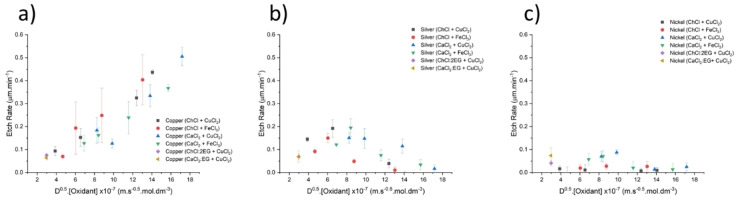
Etch depth plotted as a function of viscosity (left) and diffusion coefficient of the oxidising agent (right), for copper (a), silver (b), and nickel (c). Temperature=25 °C, time=40 minutes, stirring=100 rpm, etchant concentration=0.1 mol dm^−3^. Error bars are from the average of three measurements.

Etching rate data in Figure 4 shows that the oxidation of copper follows the predicted trend of greater etching rates in systems with lower viscosity. The two oxidising agents demonstrate similar etch rates despite their differences in redox potentials. The cation of the salt and the type of HBD present do not appear to take part directly in the etching process. This shows the validity of treating these liquids as a continuum of properties across all concentrated ionic fluids.

Silver etching data shows a curved profile with respect to viscosity. In concentrated ionic fluids (low diffusion coefficient) the etch rates for silver are similar to those for copper showing that again that mass transport controls etch rates. As viscosity decreases, silver etching increases, up to a maximum etching rate at ca. 5–10 mPa s. Below this, etching rate decreases due to the high water (>30 mol water per kg of brine) and low chloride concentrations, resulting in poor solubility of the Ag^+^ ions and the formation of AgCl precipitates. This effect of water content was also observed during the oxidation of silver in aqueous ChCl : 2EG using iodine.^[11]^

Nickel etching does not etch significantly in any of the brines or DESs, due to the propensity for nickel to passivate in the presence of water. Therefore, it would be expected that if this passivation layer is continually removed, e.g. via ultrasound, metal etching should take place at a much greater rate and show a correlation to the ease of mass transport of the oxidising species.

**Figure 5:**
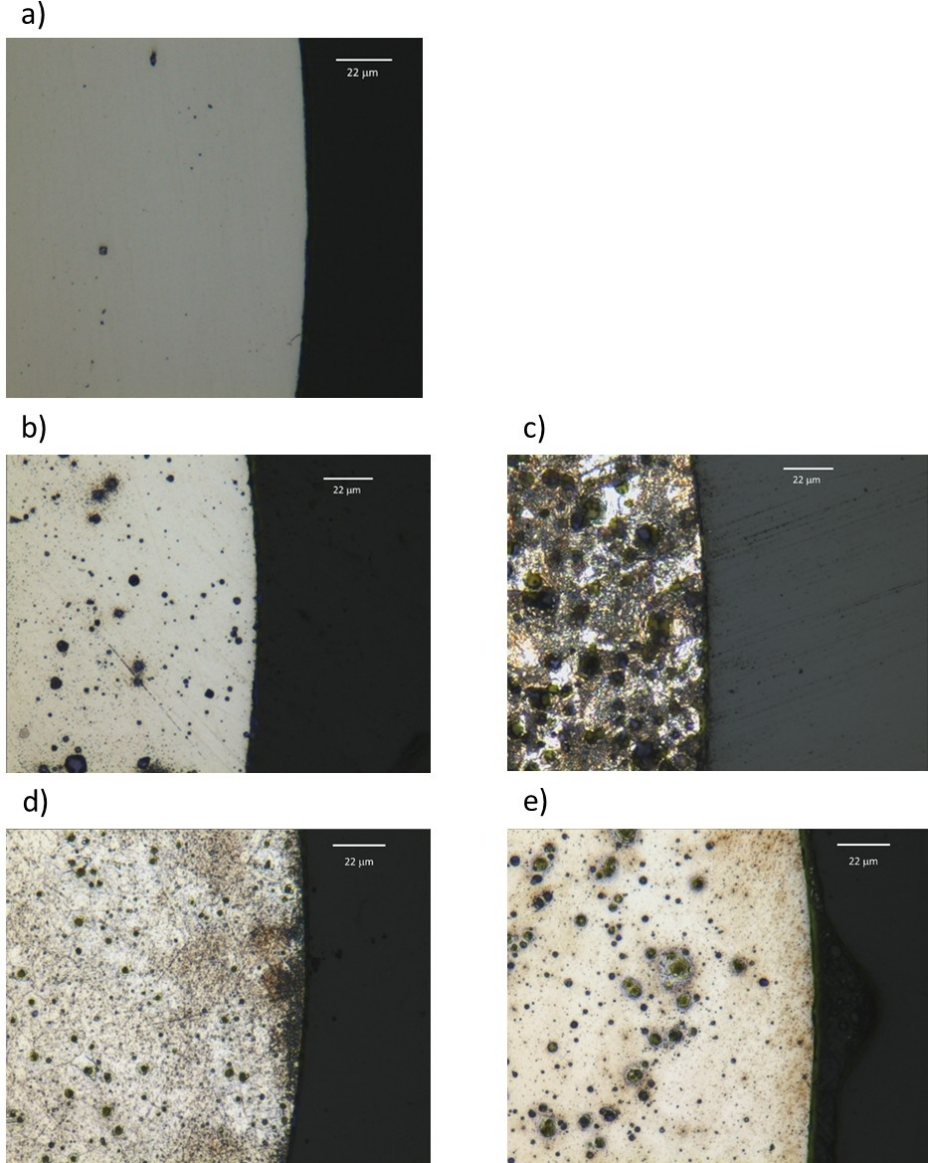
a) A representative nickel surface pre etch. Nickel surfaces after a 40 minute etch in b) 1 : 3 ChCl brine using 0.1 M FeCl_3_ etchant, c) 1 : 3 CaCl_2_ brine using 0.1 M FeCl_3_ etchant, d) ChCl : 2EG using 0.1 M FeCl_3_ etchant and e) CaCl_2_ : EG using 0.1 M FeCl_3_ etchant. The darker area on the right‐hand side of each image is the epoxy substrate.

2D images of selected nickel wires after etching are shown in Figure 5. Pitting of the surface can be seen across all samples. The ChCl brine and ChCl : 2EG (Figure 5b and d) produces smaller pits than the corresponding CaCl_2_ brine and CaCl_2_ : EG (Figure 5c and e). Surface passivation, due to low mass transport and the formation of supersaturated layers forming close to the metal surface, can be seen occurring in both DES samples and the CaCl_2_ brines. This shows the importance of understanding the relative activity of Cl^−^ and H_2_O in these systems.

This suggests that DESs with water added or concentrated brines (where the halide concentration >1 mol/kg) can be used interchangeably but water and chloride activity can affect the behaviour of metals when carried out under static conditions. This can be advantageous for selective metal dissolution but the often‐raised issue of slow mass transport with DESs can be seen to be always rate limiting. It has recently been shown that forced mass transport using laminar flow, jets or ultrasound can circumvent these issues and lead to very rapid rates of metal processing.^[41]^

## Conclusions

This study proposed that there is a continuum of properties across concentrated ionic fluids meaning that concentrated brines could be treated in the same way as deep eutectic solvents. It was shown that the molar conductivity and viscosity of calcium chloride and choline chloride brines and DESs demonstrated this continuum showing that viscosity is all that affects charge transport in the investigated systems.

The diffusion coefficients of copper and iron chloro‐complexes were determined using cyclic voltammetry via the Randles–Sevcik equation. It was shown that the diffusion coefficients for both species were very similar and diffusion coefficients for these species were again only controlled by the fluidity of the fluid, again confirming the validity of a continuum model. Application of Cu^II^ and Fe^III^ as oxidising agents to etch copper, silver, and nickel showed that dissolution rates of copper were the same for both etchants and controlled only by diffusion. The ability to tune the chemical properties of the liquid depending upon the activity of water and chloride was demonstrated for silver etching where dissolution rates could be decreased by adding more than 20 wt % water (molar ratio DES: 1.7H_2_O) due to the poor solubility of Ag^I^ complexes in the presence of chloride ions. This selectivity was further demonstrated for nickel etching where even a relatively small concentration of water resulted in the formation of passivation layers. This study has shown that a continuum model of properties can be used across concentrated ionic fluids to show that concentrated brines behave in a similar way to DESs. However, when processing metals, specific metal‐water interactions can enable selective dissolution. Subsequent work will show that passivation can be overcome using forced mass transport and DESs and brines can be used interchangeably.

## Supporting Information

The DESs were prepared by mixing the components in the desired molar ratio and heating until a homogeneous liquid had formed, and water was then added to form the correct water content. Brines were prepared by dissolving the respective chloride salts in water at the correct molar ratios. The full experimental details are described in the Supporting Information. Conductivity, viscosity, and density were recorded, along with cyclic voltammograms of the Fe^III/II^ and Cu^II/I^ redox couples at different scan rates. This data is also available in the Supporting Information, along with the corresponding Randles–Sevcik plots and an extended Walden plot comparing experimental data with literature values.[^35^, ^39^, ^42^, ^43^, ^44^, ^45^]

## Conflict of interest

The authors declare no conflict of interest.

1

## Supporting information

As a service to our authors and readers, this journal provides supporting information supplied by the authors. Such materials are peer reviewed and may be re‐organized for online delivery, but are not copy‐edited or typeset. Technical support issues arising from supporting information (other than missing files) should be addressed to the authors.

Supporting Information

## Data Availability

The data that support the findings of this study are available from the corresponding author upon reasonable request.
