# Supplementary material for: Concentrated Ionic Fluids: Is There a Difference Between Chloride‐Based Brines and Deep Eutectic Solvents?
Source: Angew Chem Int Ed Engl. 2023 Oct 11;62(46):e202311140. doi: 10.1002/anie.202311140 (PMC10953321; doi:10.1002/anie.202311140)
Supplement: Supplementary file 1 — Supporting Information [file ANIE-62-0-s001.pdf]

Angewandte Chemie
Eine Zeitschrift der Gesellschaft Deutscher Chemiker

Supporting Information

Concentrated Ionic Fluids: Is There a Difference Between Chloride-Based Brines and Deep Eutectic Solvents?

*G. Zante, C. E. Elgar, K. George, A. P. Abbott, J. M. Hartley**

Experimental Section

Reagents and solvent preparation

Aqueous chloride brines were prepared by dissolving choline chloride (ChCl, Sigma Aldrich, >98%) or calcium chloride hexahydrate ($CaCl_2{\cdot}6H_2O$, Honeywell, >97%) in deionised water (Elga Purelab Option apparatus) at varying molar ratios of salt-to-water (1:3, 1:4, 1:5, 1:10, 1:20 and 1:50). The mixtures were stirred at a temperature of 50 °C until clear and homogeneous liquids were obtained. The DES ChCl:2EG was made by mixing and heating choline chloride (ChCl) (Scientific Laboratory Supplies, 99%) with ethylene glycol (EG) (98%, Sigma Aldrich) in a 1:2 molar ratio at 80 °C until a homogenous liquid had formed. Aqueous solutions of ChCl:2EG were made by adding different weight % of water (10 to 40 wt%) to the DES. The system of 1:1 molar ratio of calcium chloride hexahydrate and ethylene glycol was made by mixing and heating the two components at 50 °C until a homogenous liquid had formed. Solutions containing oxidising agents were prepared by dissolving 0.1 mol dm^{-3} of anhydrous copper(II) chloride (Alfa Aesar, 99.9%), or iron(III) chloride (Acros Organics, 99%) in the relevant solvent.

Instrumentation

The viscosity values of the different brines were measured at 25 °C using a Seiko EG&G QCM922A Quartz Crystal Microbalance (QCM), according to previously described procedures.[33] Solvent densities were estimated by weighing 100 mL of the solvent heated at 25 °C in a 100 mL volumetric flask, using a 4 d.p. balance (Mettler Toledo). Conductivity values were recorded using a commercial chemical-resistant type conductivity probe (SC72SN-31-AA; Yokogawa) and metre (SC72 Personal Conductivity Meter; Yokogawa), with an integrated temperature sensor (accuracy within ± 0.7 °C). The cell constant for the commercial conductivity probe was 5.2 ± 0.5 cm^{-1}, and conductivity standards (certified traceable to NIST; VWR) of 44,479, 11,419, 8,863, 1,249, 885, 442, and 74 µS cm^{-1} (at 19 °C) were used as received to perform calibrations. The reported conductivity values are an average of at least three repeat measurements.

The electrochemical behaviour of the target metals was studied by cyclic voltammetry (CV), using an IVIUMnSTAT potentiostat. A 0.5 mm diameter platinum disk was used as the working electrode, with a platinum flag counter electrode, and a 3.0 mol dm^{-3} KCl Ag/AgCl reference electrode. Prior to each experiment, the working electrode was polished with a 0.3 µm and 0.05 µm alumina slurry, rinsed with deionised water, and air dried. The reference electrode potential was calibrated to the $[Fe(CN)_6]^{3-/4-}$ redox couple, which is used as an internal

standard to ensure comparability between the different solvents. The scan rates were 5 to 100 mV s^{-1}. Diffusion coefficients, *D*, of the soluble copper and iron species in the eight different brines were determined via the Randles-Sevcik equation (**Eq. 1**):

$$i_p = 0.4463nFAC(\frac{nFvD}{RT})^{\frac{1}{2}} \quad (1)$$

where i_p is measured current, *A* is electrode area, *C* is concentration, *F* is the Faraday constant, *n* is number of electrons, v is scan rate, *R* is gas constant, and *T* is temperature.

Etching of copper, silver, and nickel wires

Etching was carried out on copper (dia. = 1.25 mm), silver (dia. = 0.5 mm), and nickel (dia. = 1.25 mm) wires, using a solution of 0.1 mol dm^{-3} $CuCl_2$ or $FeCl_3$ in the different chloride brines and aqueous ChCl:2EG systems, at a temperature of 25 °C, over the course of 40 minutes, with stirring of 100 rpm. The resin blocks were mounted in the solution with the wire faces parallel to the vortex of the stirrer bar, which was 1 cm away from the sample. Samples were washed using deionised water, blotted dry using lint-free tissues, and the etch depth was analysed using a Zeta Instruments Zeta 2000 optical profiler using the inbuilt Zeta3D software version 1.8.5. Cross-sectional line profiles were measured across the surface, and the etch depth was recorded against the insoluble epoxy resin as a reference level. Using the known diameter of the wire and the calculated etching depth, combined with the density of the metal, the dissolution rate was determined. Between each experiment the blocks were polished using SiC abrasive paper down to 1200 grit, followed by polishing with diamond paste (6 and 1 µm).

Additional tables and figures

Table S1. Physicochemical properties of the brines and aqueous DES at 25 °C, and calculated theoretical chloride and water concentrations per kg of solvent. Uncertainties in the last digit are in brackets.

Aqueous ChCl:2EG					
**DES + water wt%**	**Chloride conc. / mol kg^{-1}**	**HBD conc. / mol kg^{-1}**	**Viscosity / mPa s**	**Density / g cm^{-3}**	**Conductivity / mS cm^{-1}**
ChCl: 2EG	3.79	7.6	37 *	1.12 *	7.61 *
ChCl: 2EG, 10 wt% H_2O	3.41	12.4	18.4(6)	1.093(4)	14.5(4)
ChCl: 2EG, 20 wt% H_2O	3.03	17.2	10.13(6)	1.085(7)	22.9(6)
ChCl: 2EG, 30 wt% H_2O	2.65	22.0	6.3(2)	1.074(2)	32.1(2)
ChCl: 2EG, 40 wt% H_2O	2.27	26.8	4.35(2)	1.051(5)	40.9(6)
Choline chloride brines					
**Brine / molar ratio**	**Chloride conc. / mol kg^{-1}**	**Water conc. / mol kg^{-1}**	**Viscosity / mPa s**	**Density / g cm^{-3}**	**Conductivity / mS cm^{-1}**
ChCl: $3H_2O$	5.16	15.5	14.08(6)	1.097(4)	43.3(3)
ChCl: $4H_2O$	4.73	18.9	8.0(1)	1.078(3)	57.7(1)
ChCl: $5H_2O$	4.35	21.8	6.24(9)	1.090(1)	67.6(5)
ChCl: $10H_2O$	3.13	31.3	4.56(2)	1.067(1)	96.3(2)
ChCl: $20H_2O$	2.00	40.0	1.79(2)	1.047(4)	92(2)
ChCl: $50H_2O$	0.96	48.1	1.327(5)	1.022(3)	59.7(4)
Calcium chloride brines					
**Brine / molar ratio**	**Chloride conc. / mol kg^{-1}**	**Water conc. / mol kg^{-1}**	**Viscosity / mPa s**	**Density / g cm^{-3}**	**Conductivity / mS cm^{-1}**
$CaCl_2 \cdot 6H_2O$: $3H_2O$	7.32	33.0	7.72(8)	1.385(1)	113.7(4)
$CaCl_2 \cdot 6H_2O$: $4H_2O$	6.87	34.4	6.11(5)	1.356(5)	134.5(5)

Table S1. Physicochemical properties of the brines and aqueous DES at 25 °C, and calculated theoretical chloride and water concentrations per kg of solvent. Uncertainties in the last digit are in brackets.

$CaCl_2·6H_2O$: $5H_2O$	6.47	35.6	4.14(7)	1.359(3)	144.6(9)
$CaCl_2·6H_2O$: $10H_2O$	5.01	40.1	2.58(3)	1.247(2)	189.7(4)
$CaCl_2·6H_2O$: $20H_2O$	3.45	44.9	2.09(9)	1.189(1)	182(2)
$CaCl_2·6H_2O$: $50H_2O$	1.79	50.0	1.26(7)	1.081(4)	119.0(2)

* Data taken from ref 33.

Table S2. Diffusion coefficients of Cu^{I}, Cu^{II}, Fe^{II}, and Fe^{III} in the different chloride brines and in aqueous ChCl:2EG. Uncertainties in the last digit are in brackets.

Aqueous ChCl:2EG				
**Chloride content / mol kg^{-1}**	**D (Cu^{I}) / cm^2 s^{-1}**	**D (Cu^{II}) / cm^2 s^{-1}**	**D (Fe^{II}) / cm^2 s^{-1}**	**D (Fe^{III}) / cm^2 s^{-1}**
3.79 *	8.57 x 10^{-8}	8.90 x 10^{-8}	1.44(1) x 10^{-7}	1.51(1) x 10^{-7}
3.41	1.65(3) x 10^{-7}	1.48(1) x 10^{-7}	1.55(1) x 10^{-7}	1.61(2) x 10^{-7}
3.03	3.07(2) x 10^{-7}	2.78(3) x 10^{-7}	2.010(3) x 10^{-7}	2.24(2) x 10^{-7}
2.65	4.99(7) x 10^{-7}	4.24(5) x 10^{-7}	3.14(2) x 10^{-7}	3.46(2) x 10^{-7}
2.27	4.83(6) x 10^{-7}	7.37(4) x 10^{-7}	4.58(3) x 10^{-7}	4.77(6) x 10^{-7}

Choline chloride brines				
**Chloride content / mol kg^{-1}**	**D (Cu^{I}) / cm^2 s^{-1}**	**D (Cu^{II}) / cm^2 s^{-1}**	**D (Fe^{II}) / cm^2 s^{-1}**	**D (Fe^{III}) / cm^2 s^{-1}**
5.16	1.550(9) x 10^{-7}	1.51(1) x 10^{-7}	2.04(3) x 10^{-7}	2.18(2) x 10^{-7}
4.73	4.35(5) x 10^{-7}	4.28(5) x 10^{-7}	3.47(6) x 10^{-7}	3.64(2) x 10^{-7}
4.35	7.90(6) x 10^{-7}	7.73(4) x 10^{-7}	4.90(3) x 10^{-7}	4.9(1) x 10^{-6}
3.13	1.61(2) x 10^{-6}	1.54(2) x 10^{-6}	5.73(6) x 10^{-7}	7.68(4) x 10^{-6}
2.00	2.4(2) x 10^{-6}	1.91(8) x 10^{-6}	1.37(6) x 10^{-6}	1.96(3) x 10^{-6}
0.96	2.2(2) x 10^{-6}	1.98(7) x 10^{-6}	1.12(6) x 10^{-6}	1.7(2) x 10^{-6}

Calcium chloride brines				
**Chloride content / mol kg^{-1}**	**D (Cu^{I}) / cm^2 s^{-1}**	**D (Cu^{II}) / cm^2 s^{-1}**	**D (Fe^{II}) / cm^2 s^{-1}**	**D (Fe^{III}) / cm^2 s^{-1}**
7.32	6.09(2) x 10^{-7}	6.80(5) x 10^{-7}	4.57(2) x 10^{-7}	4.78(3) x 10^{-7}
6.87	9.96(5) x 10^{-7}	9.71(5) x 10^{-7}	7.08(1) x 10^{-7}	7.077(3) x 10^{-7}

Table S2. Diffusion coefficients of Cu^{I}, Cu^{II}, Fe^{II}, and Fe^{III} in the different chloride brines and in aqueous ChCl:2EG. Uncertainties in the last digit are in brackets.

6.47	1.157(7) x 10^{-6}	1.14(3) x 10^{-6}	9.66(3) x 10^{-7}	1.0(1) x 10^{-7}
5.01	1.8(1) x 10^{-6}	1.91(8) x 10^{-6}	1.24(2) x 10^{-6}	1.34(5) x 10^{-6}
3.45	2.7(8) x 10^{-6}	2.6(1) x 10^{-6}	2.13(2) x 10^{-6}	2.1(2) x 10^{-6}
1.79	3.3(4) x 10^{-6}	3.0(3) x 10^{-6}	2.36(5) x 10^{-6}	2.47(7) x 10^{-6}

* Values obtained from ref 33.

a) 10 wt% water

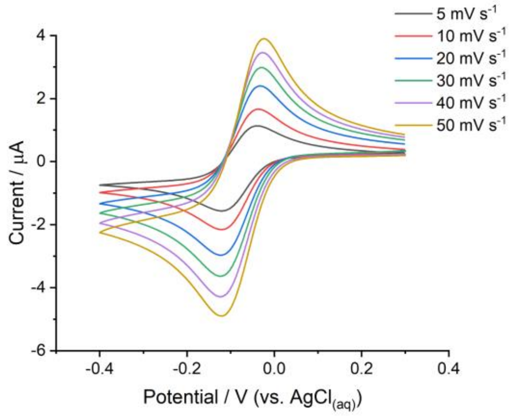

b) 20 wt% water

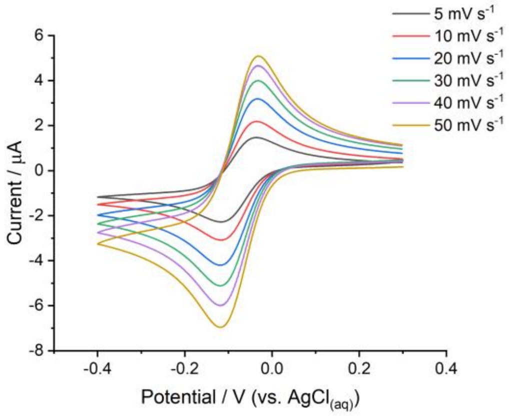

c) 30 wt% water

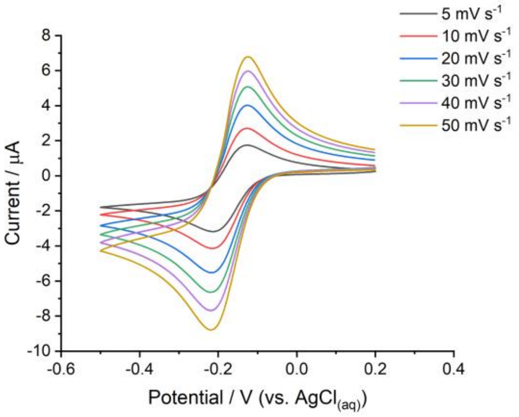

d) 40 wt% water

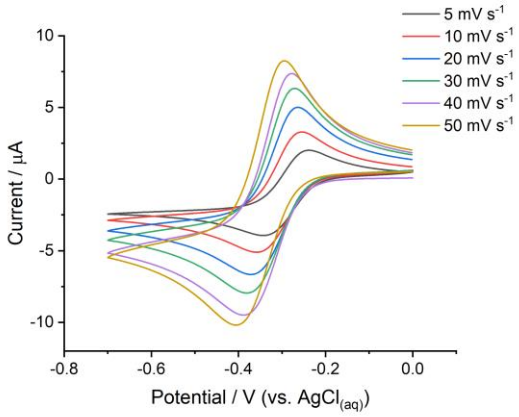

Figure S1. CVs at different scan rates for copper(II) chloride in aqueous ChCl:2EG, measured at a Pt-disc working electrode, vs a 3.0 mol dm^{-3} KCl Ag/AgCl reference electrode. Scans recorded at room temperature. The first scan is presented here.

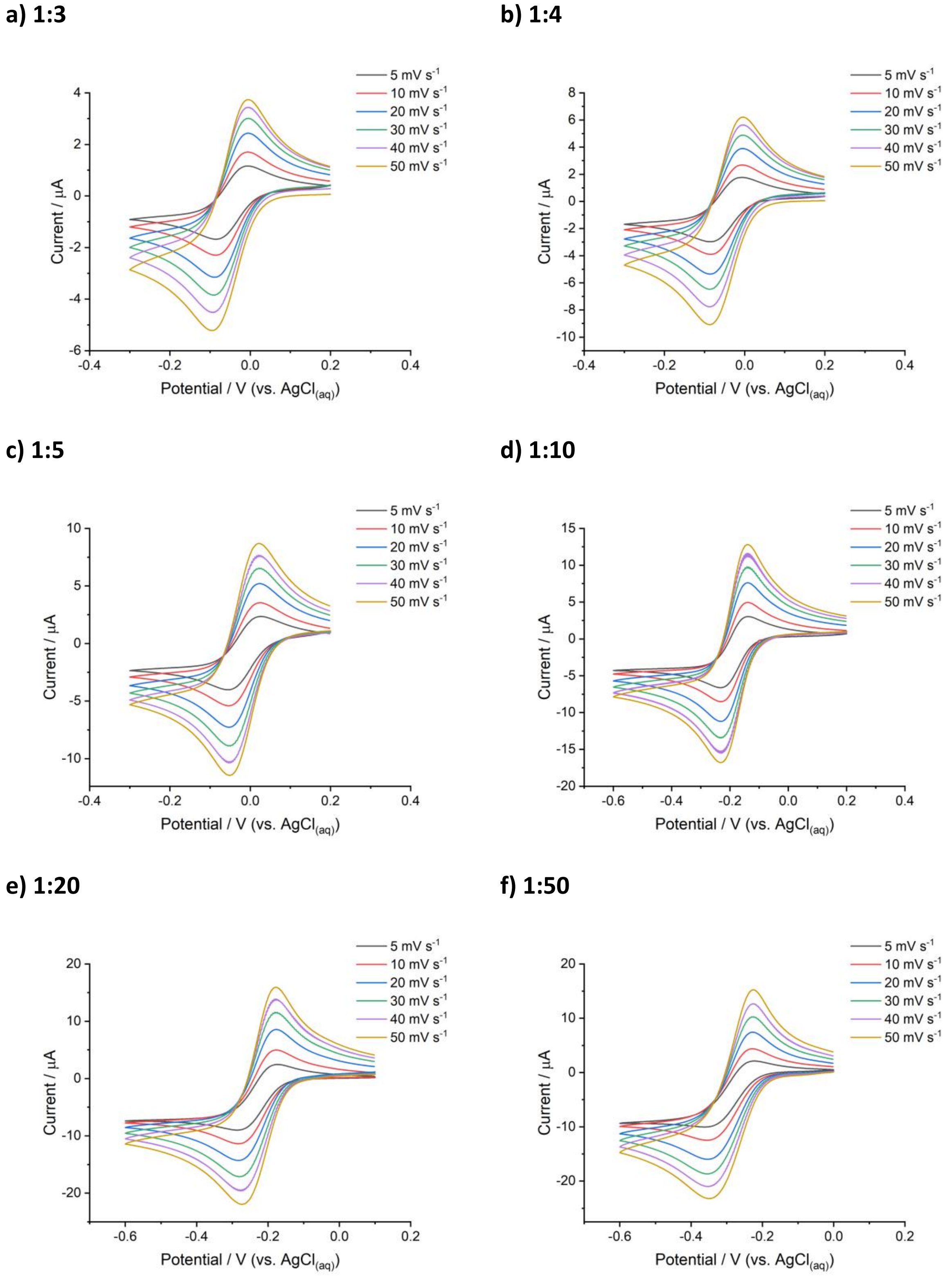

Figure S2. CVs at different scan rates for copper(II) chloride in choline chloride brines, measured at a Pt-disc working electrode, vs a 3.0 mol dm^{-3} KCl Ag/AgCl reference electrode. Scans recorded at room temperature. The first scan is presented here.

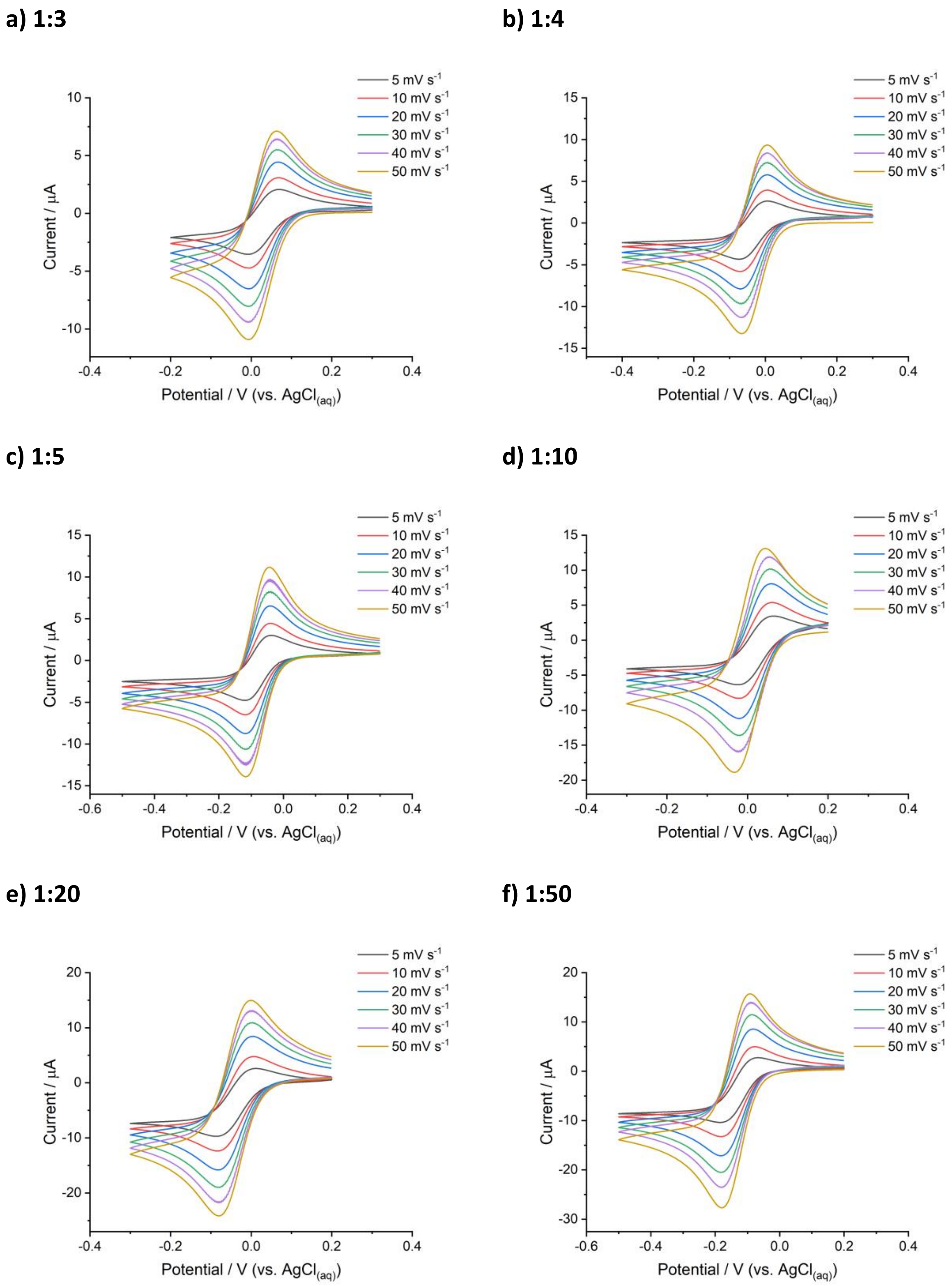

Figure S3. CVs at different scan rates for copper(II) chloride in calcium chloride brines, measured at a Pt-disc working electrode, vs a 3.0 mol dm^{-3} KCl Ag/AgCl reference electrode. Scans recorded at room temperature. The first scan is presented here.

a) 10 wt% water

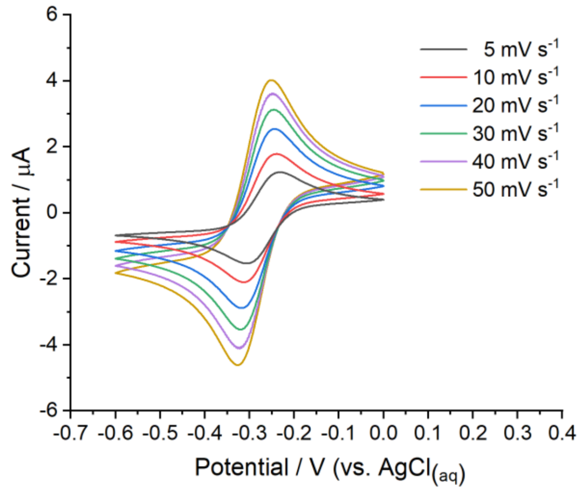

b) 20 wt% water

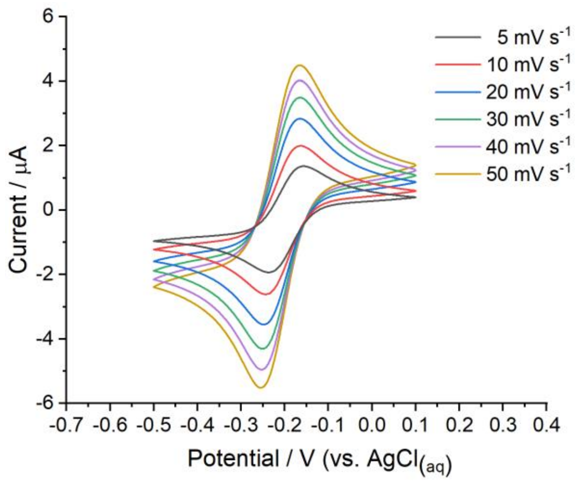

c) 30 wt% water

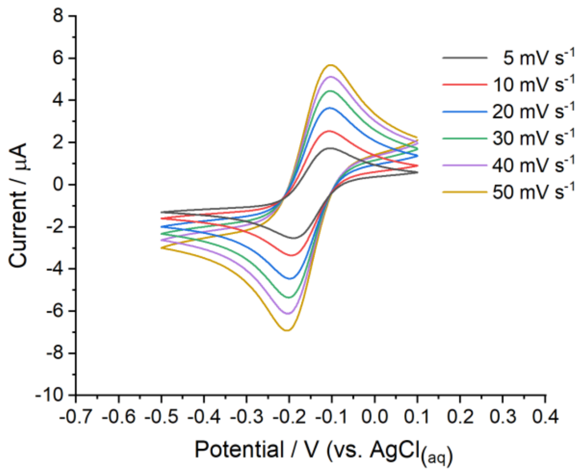

d) 40 wt% water

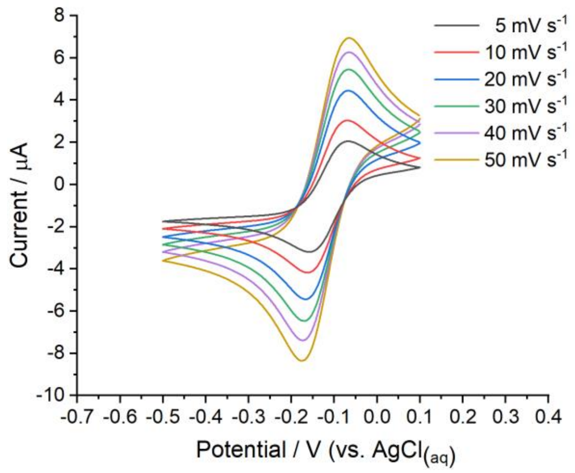

Figure S4. CVs at different scan rates for iron(III) chloride in aqueous ChCl:2EG, measured at a Pt-disc working electrode, vs a 3.0 mol dm^{-3} KCl Ag/AgCl reference electrode. Scans recorded at room temperature. The first scan is presented here.

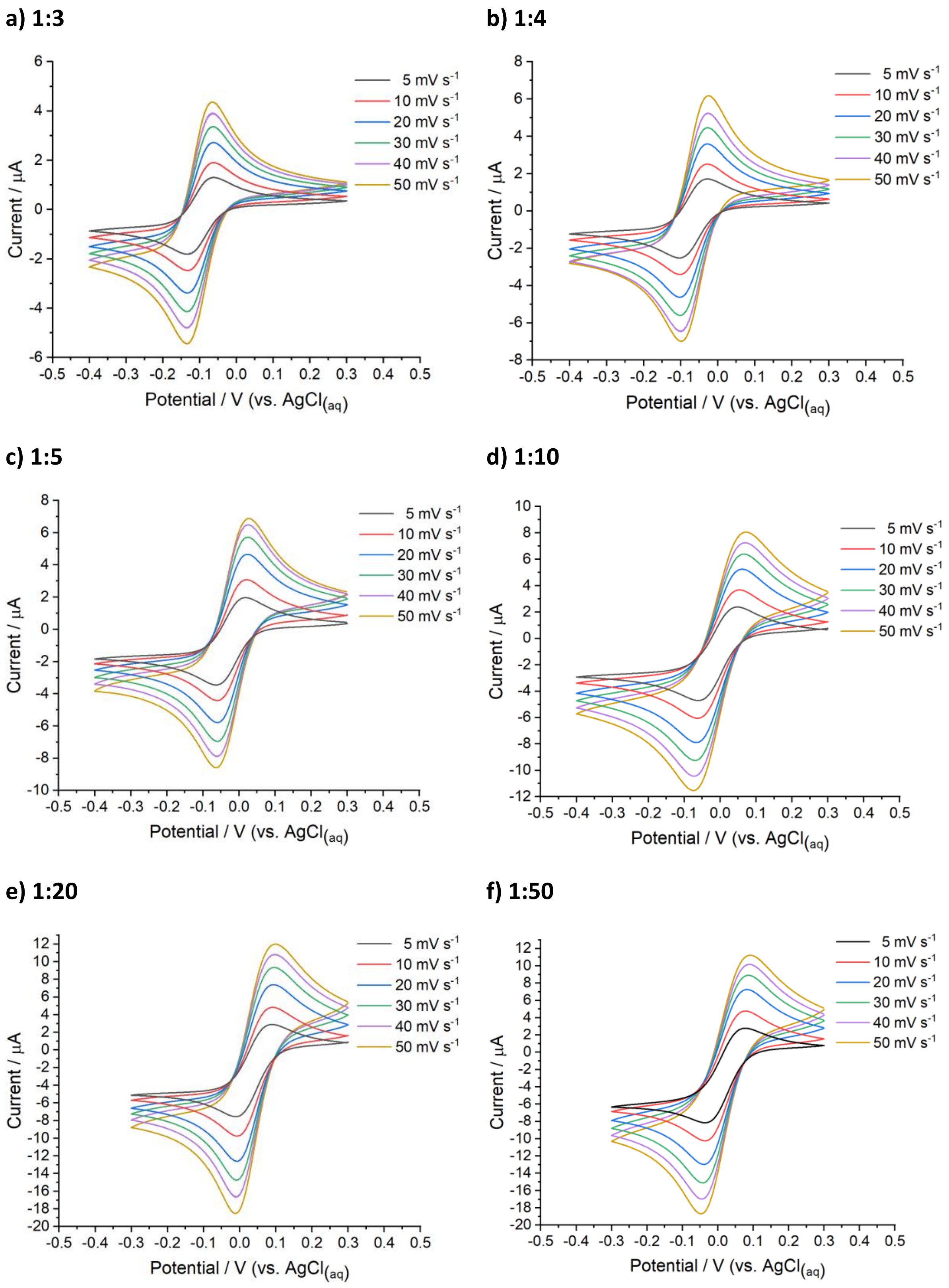

Figure S5. CVs at different scan rates for iron(III) chloride in choline chloride brines, measured at a Pt-disc working electrode, vs a 3.0 mol dm^{-3} KCl Ag/AgCl reference electrode. Scans recorded at room temperature. The first scan is presented here.

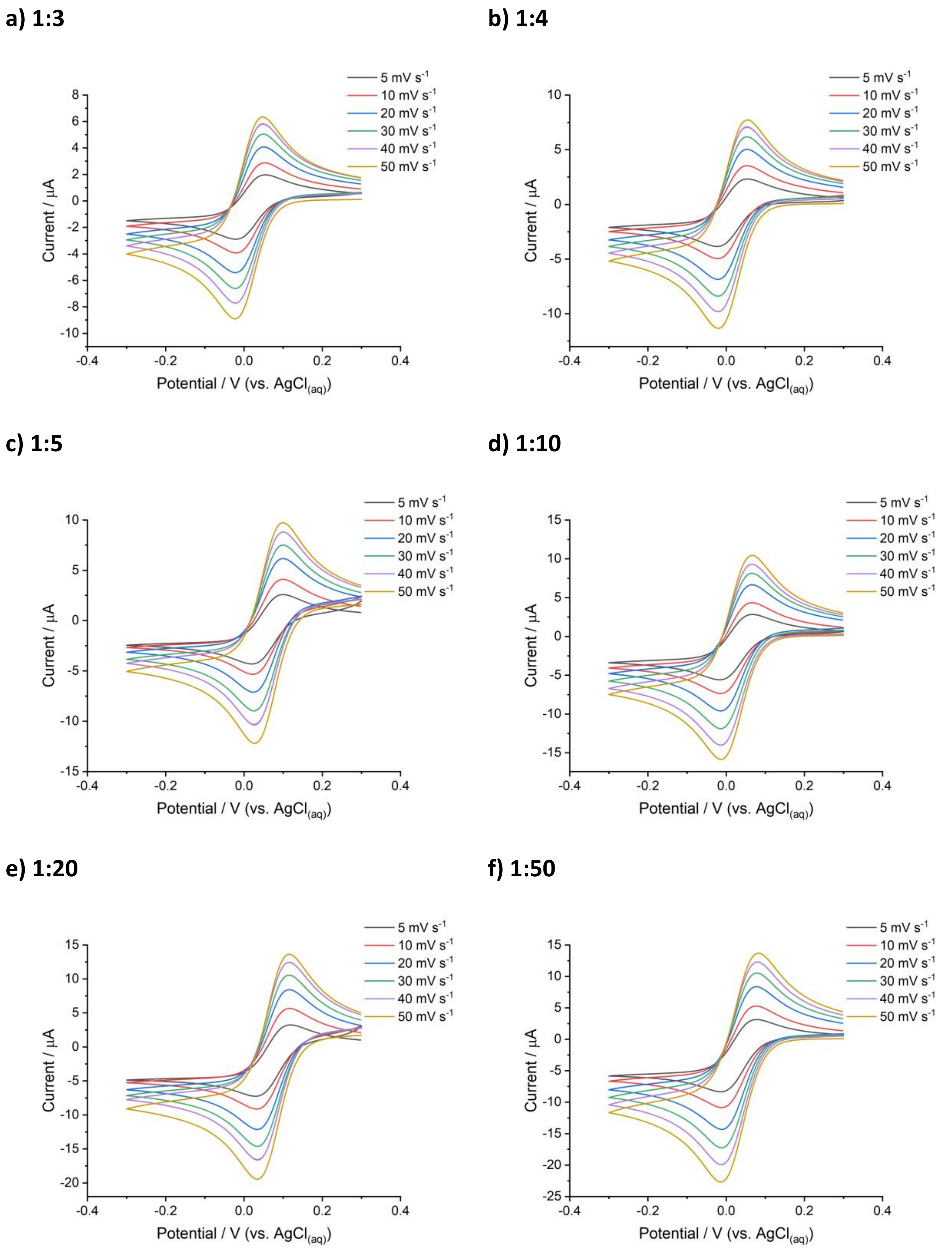

Figure S6. CVs at different scan rates for iron(III) chloride in calcium chloride brines, measured at a Pt-disc working electrode, vs a 3.0 mol dm^{-3} KCl Ag/AgCl reference electrode. Scans recorded at room temperature. The first scan is presented here.

a) Cu in aq. ChCl: 2EG

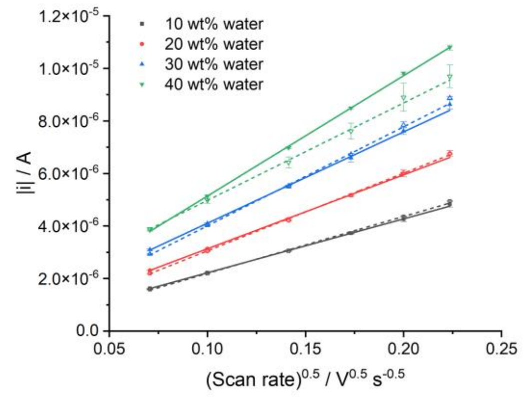

b) Fe in aq. ChCl: 2EG

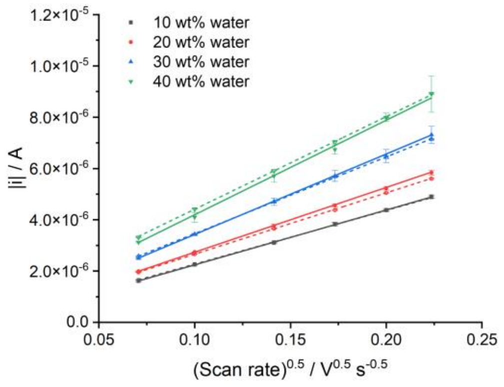

c) Cu in ChCl brine

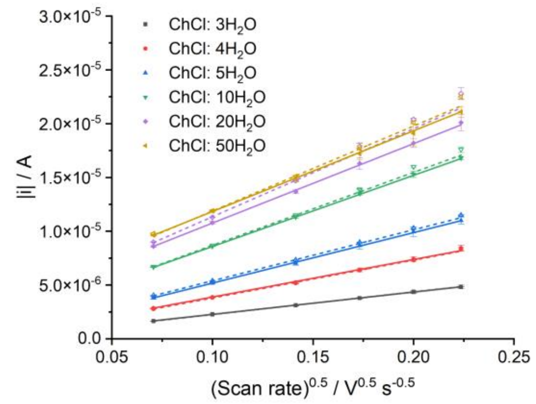

d) Fe in ChCl brine

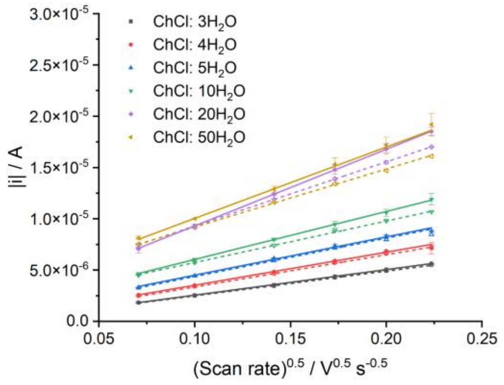

e) Cu in CaCl2 brine

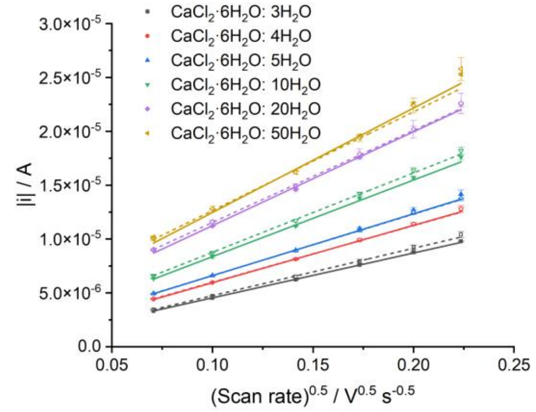

f) Fe in CaCl2 brine

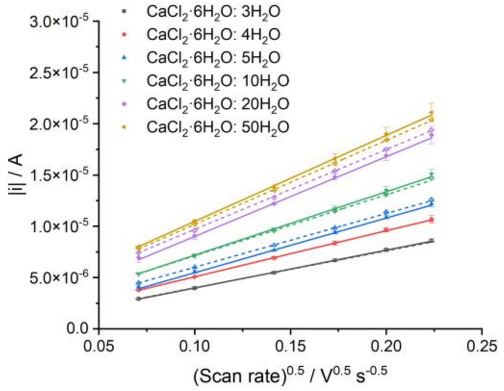

Figure S7. Randles-Sevcik plots for copper ions (left) and iron ions (right) in aqueous ChCl:2EG (a,b), choline chloride brines (c,d), and calcium chloride brines (e,f). Filled symbols are i_{pc}, hollow symbols are i_{pa}. Lines are best fit.

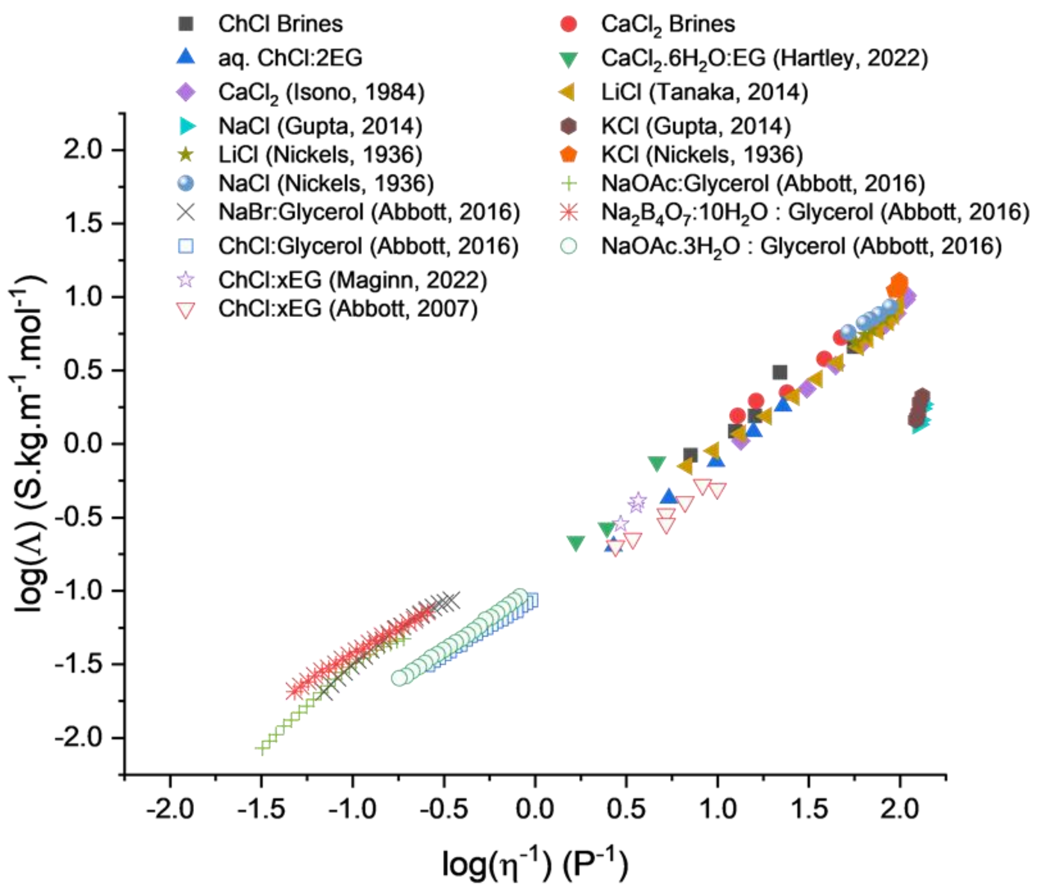

Figure S8. Walden plot of molar conductivity as a function of fluidity for solvents used in this work compared to literature values. Temperature is 25 °C. Literature values from refs 33, 37, 40-43.

References

[33] T. Isono, *J. Chem. Eng. Data* **1984**, *29*, 45-52.
[37] J. M. Hartley, J. Allen, J. Meierl, A. Schmidt, I. Krossing, A. P. Abbott, *Electrochim. Acta* **2022**, *402*, 139560.
[40] L. Nickels, A. J. Allmand, *J. Phys. Chem.* **1937**, *41*, 861-872.
[41] K. Tanaka, R. Tamamushi, *Zeitschrift für Naturforschung A* **1991**, *46*, 141-147.
[42] A. P. Abbott, C. D'Agostino, S. J. Davis, L. F. Gladden, M. D. Mantle, *Phys Chem Chem Phys* **2016**, *18*, 25528-25537.
[43] V. Gupta, A. K. Sharma, M. Sharma, *Chemical Science Transactions* **2014**, *3*, 763-744.
